# The angiographic presentation of European Moyamoya angiopathy

**DOI:** 10.1007/s00415-021-10684-6

**Published:** 2021-07-08

**Authors:** Sara Pilgram-Pastor, René Chapot, Markus Kraemer

**Affiliations:** 1 Department of Neuroradiology, Alfried Krupp Hospital, Essen, Germany; 2 University Institute of Diagnostic and Interventional Neuroradiology, Inselspital, University of Bern, Bern, Switzerland; 3 Department of Neurology, Medical Faculty, Heinrich-Heine University Düsseldorf, Düsseldorf, Germany; 4 Department of Neurology, Alfried Krupp Von Bohlen Und Halbach Hospital, Alfried-Krupp-Str. 21, 45117 Essen, Germany

**Keywords:** Moyamoya angiopathy, Non-Asian, European, Angiographic presentation

## Abstract

**Background and purpose:**

Little is known about the angiographic presentation of Moyamoya angiopathy (MMA) in non-Asian patients.

**Methods:**

Conventional cerebral angiograms from 155 Caucasian patients diagnosed as MMA were analyzed with respect to extracranial champagne bottle neck sign, Suzuki stages, collateral status, as well as presence of aneurysms and posterior cerebral artery stenosis.

**Results:**

In 84 of 155 angiograms, the extracranial carotid artery was visualized, in 65 of them (77.4%), a champagne bottle neck sign was noted. Of the 278 analyzable hemispheres, 13.7%,11.2%, 37.8%, 27.3%, 8.6%, and 1.4% were classified as Suzuki stage I, stage II, stage III, stage IV, stage V, and stage VI, respectively. Among 280 hemispheres, in 53 hemispheres (18.9%) isolated basal collaterals (pathway I) and in 104 hemispheres (37.1%) choroidal and pericallosal collaterals (including basal collaterals, pathway II) were found. In 74 hemispheres (26.4%) ethmoidal collaterals (pathways III), and in 17 hemispheres (6.1%) vault collaterals were visualized. Patients with higher Suzuki stages IV–VI (*p* = 0.008) and ethmoidal collaterals (*p* < 0.001) suffered more often from cerebral hemorrhage. Transient ischemic attacks occurred more frequently in patients with Suzuki stage I to III (*p* < 0.001). In 10 of 155 patients (6.5%), the angiogram revealed a cerebral aneurysm. In 13 patients (8.4%), a stenotic P1 segment of the posterior cerebral artery was found.

**Conclusions:**

This is so far the largest observational study about angiography in Caucasian European MMA patients. A comparison with Asian data indicates similarity of disease in Caucasian and Asian patients.

## Introduction

Moyamoya angiopathy (MMA) is a rare vasculopathy characterized by bilateral stenosis or occlusion of the intracranial part of the internal carotid artery as well as the anterior and middle cerebral arteries. The angiographic appearance of the compensatory collateral networks was initially compared to a puff of smoke—“Moyamoya” in Japanese—, which gave the disease its name [1]. The umbrella term “Moyamoya angiopathy” refers to both “Moyamoya syndrome” and “Moyamoya disease”. The former applies when the vasculopathy is associated with another condition, such as Down syndrome, neurofibromatosis (von Recklinghausen’s disease) or a state after bacterial meningitis or head radiation, and the latter when it is idiopathic. Genetic triggers are now known to cause those variants formerly considered to be idiopathic (Moyamoya disease). This etiology is well established in Asia, with the RNF 213 founder mutation, but is also increasingly recognized in Europe where heterogeneous mutations are reported [2]. Whereas the final pathophysiological pathways seem to be identical in Asian and European patients, as demonstrated in autopsy findings [3], it is unclear whether clinical and paraclinical presentations really differ between ethnic groups [4] or whether these data are biased due to an estimated high number of unreported or misdiagnosed cases outside Asia [5]. Disease presentation seems to occur in Asia at a younger age, with a peak around the age of 5 years, characterized by transient ischemic attacks and strokes [6]. Another age peak is seen in young adulthood where stroke and cerebral hemorrhage are common manifestations [6]. Recently, a study of 200 German patients found that 71.5% experienced transient ischemic attacks, 82% at least one stroke and 9.5% at least one cerebral hemorrhage [7]. While the disease and its management are well established in Asia [8], there is no consensus among neurologists outside Asia as to whether bypass surgery is also useful in European patients, although this has been shown in a number of studies [9, 10]. Greater familiarity with the angiographic presentation of MMA may help to diagnose the disease earlier and to avoid misdiagnoses. Moreover, comparison with Asian angiographic data may allow conclusions to be drawn from Asian treatment regimens. Therefore, this study was designed to deepen our understanding of the angiographic presentation of MMA in European patients.

## Materials and methods

Angiographic data of 155 patients diagnosed with MMA at the Alfried Krupp Hospital, Essen, Germany, were analyzed. The Heinrich-Heine University Duesseldorf ethical committee authorized the study and it was conducted according to the principles of the Declaration of Helsinki.

Patients with a non-Caucasian family background (Asian, Arabic or Romani) or with associated diseases, such as Down syndrome or von Recklinghausen’s disease, were excluded from the analysis. Most patients identified themselves as ethnically German, five as Eastern European, one as Spanish and one as Dutch. We analyzed first angiograms obtained in our center; so in almost all cases (except for four patients), the date of the angiogram is the date of first diagnosis.

**Fig. 1:**
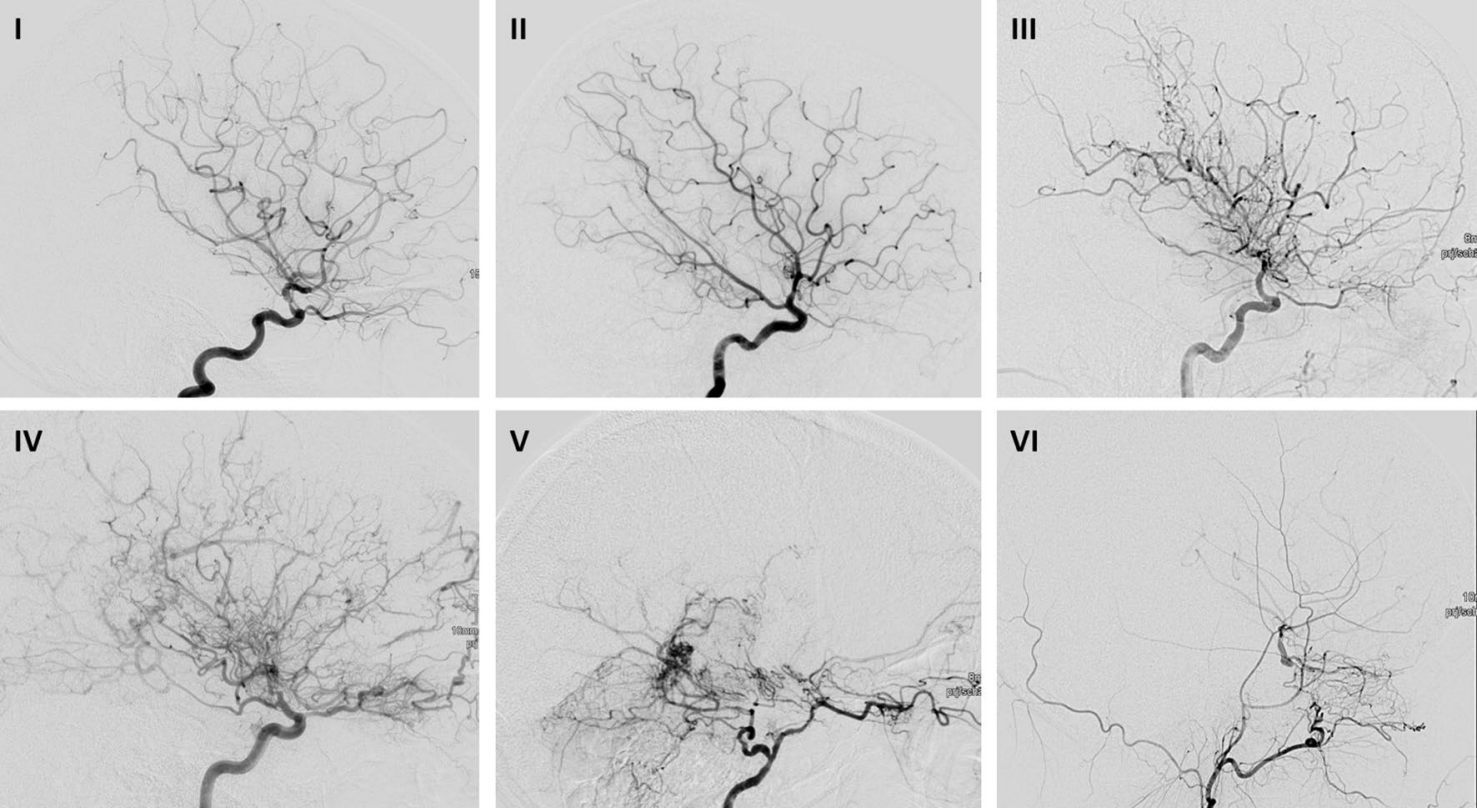
DSA images with lateral views of patients with MMD of ICA (I–V) and ECA (VI). (I) Suzuki´s Stage I: narrowing of carotid fork. (II) Suzuki´s Stage II: initiation of basal moyamoya, ACA and ACM are dilated. (III) Suzuki´s Stage III: intensification of moyamoya, remarkable moyamoya vessels at the base of the brain, MCA and ACA can be occluded. (IV) Suzuki´s Stage IV: minimization of basal moyamoya, more and more transdural anastomoses occur, next to MCA and ACA the PCA can be affected. (V) Suzuki´s Stage V: even more reduction of basal moyamoya vessels, intracerebral anastomoses between ACP and ACM occur prominent. (VI) Suzuki´s Stage VI: vascularisation of ACA and MCA exclusively through transdural anastomosis of ACE and basilar/vertebral arteries

**Fig. 2:**
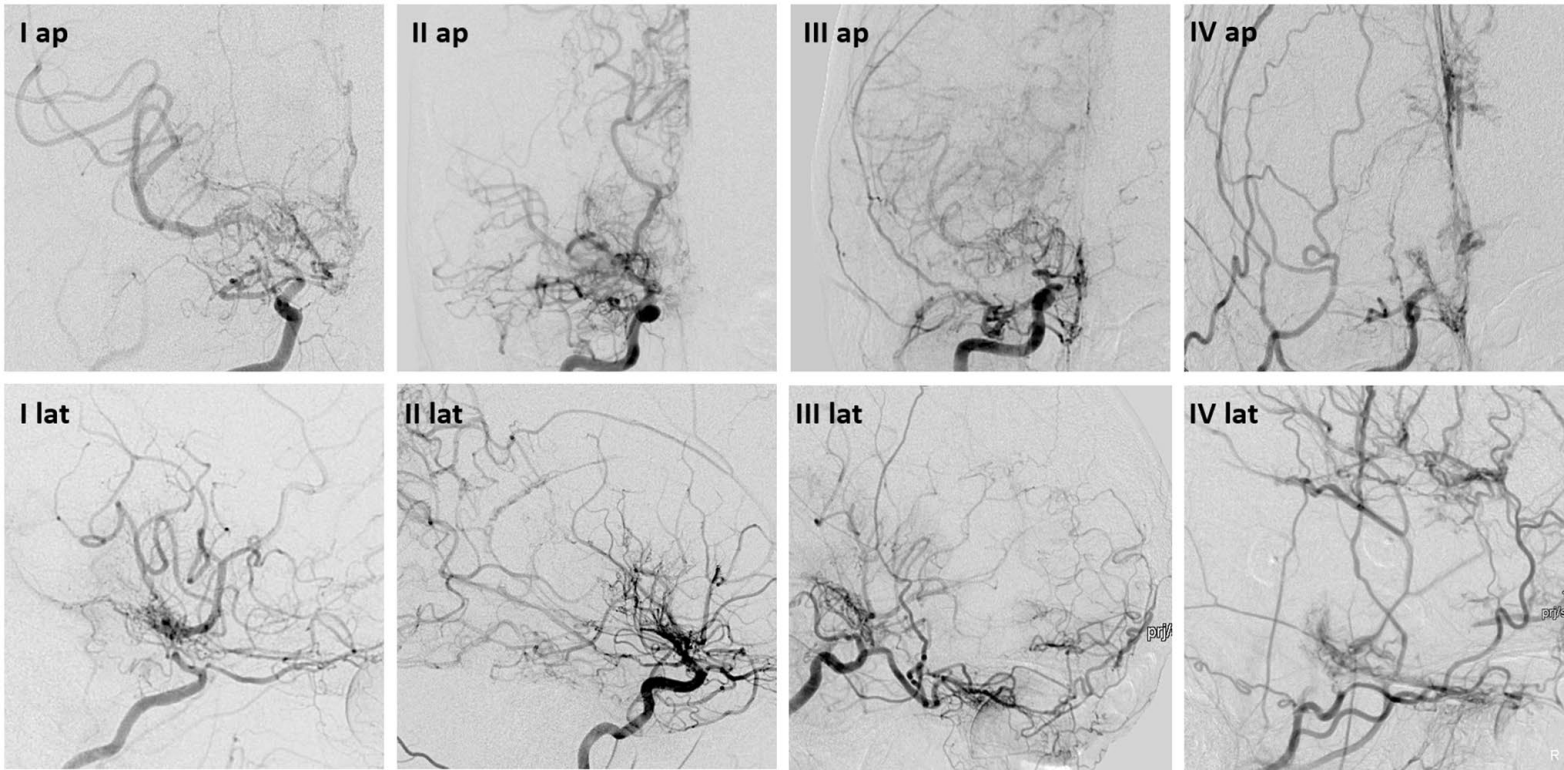
DSA images of patients with MMD of ICA (I–III) and ECA (VI) in anteroposterior (ap) and lateral (lat) views focusing on collateral pathways. (I) Suzuki and Kodama I: collateral pathway mainly through basal moyamoya vessels, (II) Suzuki and Kodama II: collateral pathway next to moyamoya vessels also through dilated choroidal and pericallosal artery supplies, (III) Suzuki and Kodama III: collateral pathway through anterior and posterior ethmoidal arteries, (IV) Suzuki and Kodama IV: collateral pathway exclusively through transdural anastomosis which is named as "Vault Moyamoya”

**Fig. 3:**
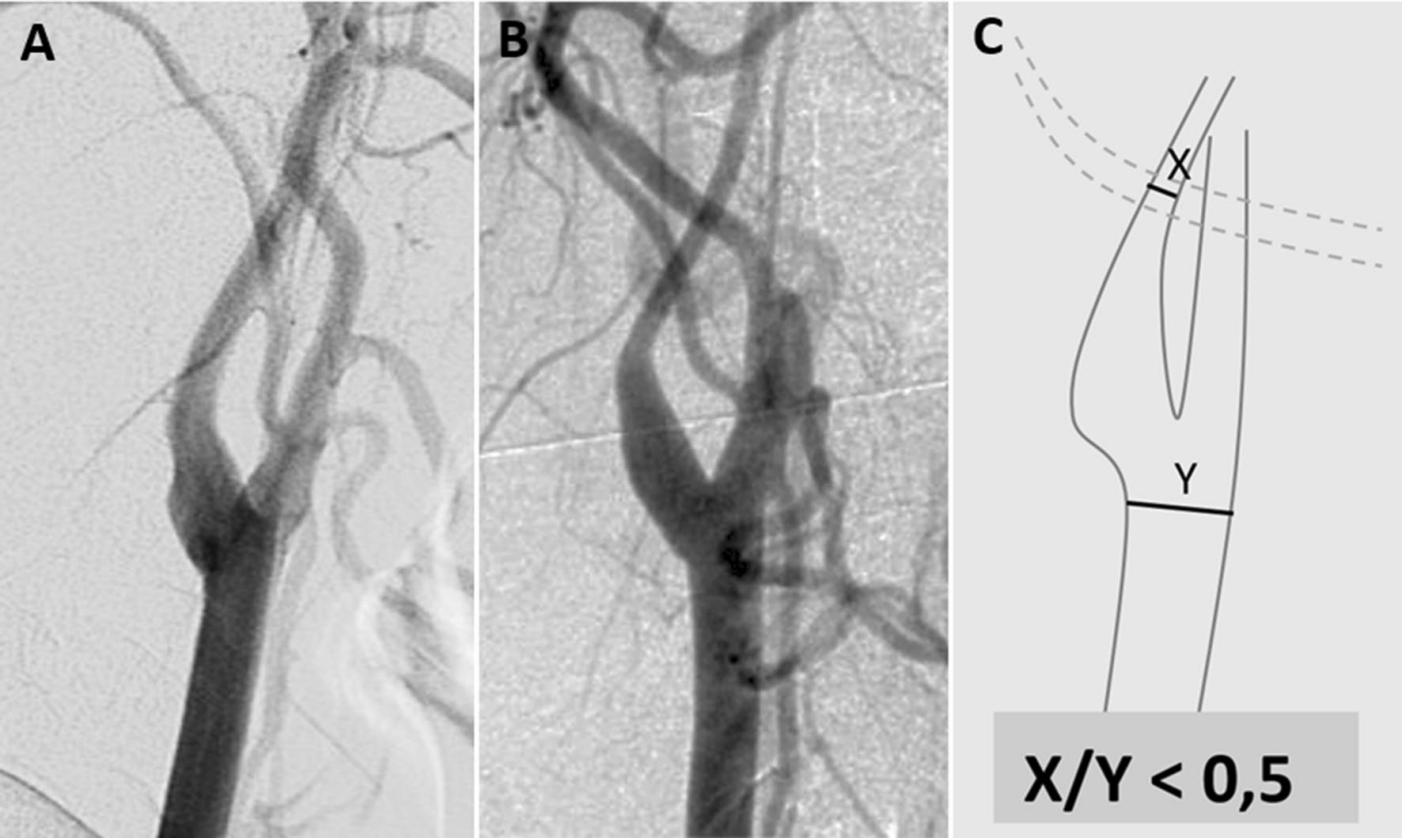
DSA images of the carotid folk in a lateral view. **A** normal angiogram of the carotid fork, **B** angiogram with a champagne bottle neck (CBN) sign which refers to a reduction in the diameter of the proximal portion of the internal carotid artery that resembles a CBN, **C** CBN in a carotid fork as scheme about where and what to measure

**Fig. 4:**
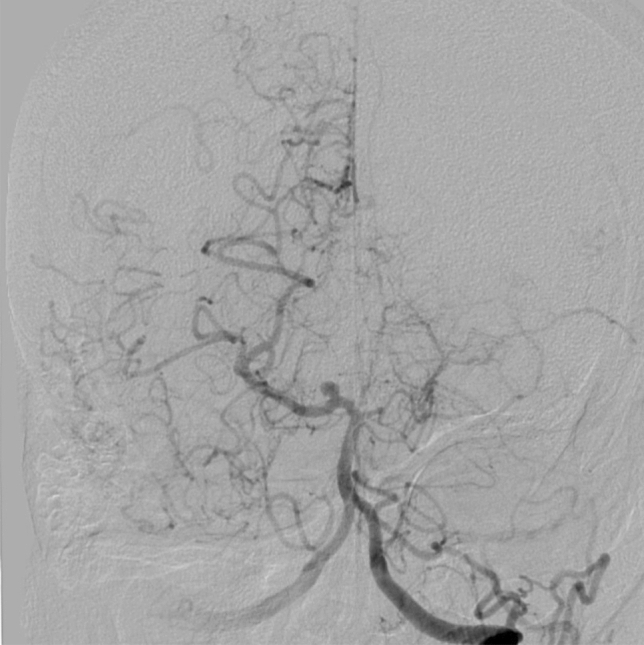
Cerebral aneurysm in Moyamoya angiopathy

Angiographic analysis was focused on the features shown in Table 1 and in Figs. 1, 2, 3, 4.

**Table 1:** Definition of features

Feature	Definition
Champagne bottle neck sign (CBN) (Fig. 1)	
CBN	More than 50% reduced diameter of the proximal portion of extracranial ICA above bulbus compared with ACC as a sign of collapsed flow in downstream stenosis or occlusion of intracranial ICA
Suzuki angiographic stages (Fig. 2)	
Suzuki stage I	“Narrowing of the carotid fork”: mild-to-moderate stenosis around ICA bifurcation with absent basal Moyamoya collateral networks
Suzuki stage II	“Initiation of the Moyamoya”: progressive narrowing of ICA, dilatation of ACA and MCA, basal Moyamoya collaterals
Suzuki stage III	“Intensification of the Moyamoya”: severe stenosis of intracranial ICA with more collaterals and non-filling of MCA and
Suzuki stage IV	“Minimization of the Moyamoya”: advanced steno-occlusive ICA, ACA and MCA with gradually enlarged collateral from the extracranial area, minimization of basal Moyamoya collaterals
Suzuki stage V	“Reduction of Moyamoya”: disappearance of ACA and MCA with increased collateral from the external carotid artery, reduction of the basal Moyamoya vessels
Suzuki stage VI	“Disappearance of the Moyamoya”: original basal Moyamoya vessels at the base of the brain are completely missing and only the collateral circulation from the external carotid artery could be seen
Modified Suzuki Score	
Modified Suzuki 0	No evidence of disease
Modified Suzuki I	Mild-to-moderate stenosis around ICA bifurcation with absent or slightly developed ICA MMDa
Modified Suzuki II	Severe stenosis around the ICA bifurcation or occlusion of either proximal anterior or MCA branches with well-developed ICA MMD
Modified Suzuki III	Occlusion of both anterior and MCA branches with well-developed ICA MMD (only a few of anterior or MCA branches or both are faintly opacified in antegrade fashion through meshwork of ICA MMD)
Modified Suzuki IV	Complete occlusion of both anterior and MCA branches with absent or small amount of ICA MMD (without opacification of either anterior or MCA branches in antegrade fashion)
Collateral pathways according to Suzuki and Kodama (Fig. 3)	
I. Basal Moyamoya	First collateral pathway with abnormal dilation of the perforating arteries, such as the lenticulo-striate artery and the thalamo-perforating artery located in the basal ganglia and thalamus
II: Choroidal and pericallosal	Second pathway with marked dilation of the anterior choroidal and posterior pericallosal
	Arteries (in patients with this pathway also basal Moyamoya is still evident)
III: Ethmoidal Moyamoya	Third pathway with dilation of the anterior and posterior ethmoidal arteries, which also function as collateral pathways, mainly from the ophthalmic arteries to the ACA branches (in patients with these collaterals the choroidal and pericallosal are still evident, the basal collaterals are markly reduced or absent)
IV: Vault collaterals	Last pathway with transdural collateral flow to pial arteries (in this stage the basal and choroidal as well pericallosal collaterals are absent)

Statistical comparisons between categorical variables were made using the chi-squared test; statistical associations were assessed using Spearman’s rank correlation coefficient. All statistical analyses were conducted with IBM SPSS Statistics version 27 (IBM Corp. Released 2020. IBM SPSS Statistics Subscription for Macintosh, Build 1.0.0.1508. Armonk, NY: IBM Corp.).

## Results

Demographic data: Out of 155 patients analyzed, 129 (83.2%) were affected bilaterally and 26 (16.8%) suffered from the unilateral variant. In 4 of the 129 bilaterally affected patients, only one hemisphere was analyzed, as the contralateral hemisphere had already been treated with bypass surgery. Altogether, 280 hemispheres were analyzed. Demographic data are shown in Table 2.

**Table 2:** Demographic data

	All	Bilateral	Unilateral
All patients, *n* (%)	155	129 (83.2%)	26 (16.8%)
Male, *n* (%)	44 (28.4%)	38 (29.5%)	6 (23.1%)
Female, *n* (%)	111 (71.6%)	91 (70.5%)	20 (76.9%)
Age of onset in years, *n* in mean (± SD)	34.1 (± 14.03)	34.5 (± 14.6)	32 (± 11.1)
Age of onset in years, median (range)	33 ()	34 ()	31 ()
Age of diagnosis in years, mean (± SD)	37.6 (± 14.3)	38.2 (± 14.7)	34 (± 11.6)
Age of diagnosis in years, median (Range)	39 ()	39 ()	34 ()
Symptoms			
TIA, *n* (%)	109 (70.3%)	91 (70.5%)	18 (69.2%)
Stroke, *n* (%)	131 (84.5%)	116 (89.9%)	15 (57.7%)
Hemorrhage, *n* (%)	16 (10.3%)	13 (10.1%)	3 (11.5%)
Headache, *n* (%)	82 (52.9%)	64 (49.6%)	18 (69.2%)
Epilepsy, *n* (%)	53 (37.2%)	48 (37.2%)	5 (19.2%)

Champagne bottle neck (CBN) sign: In 84 of 155 angiograms, the extracranial carotid artery was visualized. In 65 of these 84 angiograms (77.4%), a champagne bottle neck sign was found at least at one side. In 17 of them (20.2%), this sign was found bilaterally.

Suzuki stage: Out of 280 hemispheres analyzed, 278 could be classified in Suzuki stages: 38 (13.7%) were rated as Suzuki stage I, 31 (11.2%) as Suzuki stage II, 105 (37.8%) as Suzuki stage III, 76 (27.3%) as Suzuki stage IV, 24 (8.6%) as Suzuki stage V and 4 (1.4%) as Suzuki stage VI.

Patients between 26 and 45 years were found to have milder stages within Suzuki stage I–III compared to Suzuki stage IV–VI; by contrast, among patients older than 45 years, Suzuki stages IV–VI were significantly more common compared to the milder Suzuki stages I to III (*p* = 0.025). The age group from 0 to 15 failed to show significant differences between lower (I–III) and higher (IV–VI) Suzuki stages.

As expected due to the grading system, basal collaterals were found more frequently in the lower Suzuki stages I–III compared to the higher stages IV–VI (I-III: 52 of 53, 98.1% versus 1 of 53, 1.9%, *p* < 0.001). The same was demonstrated for choroidal and pericallosal collaterals (I–III: 90 of 104, 86.5% versus IV–VI: 14 of 104, 13.5%, *p* = 0.000). Vice versa, ethmoidal collaterals (IV–VI: 71 of 74, 95.9% versus 1–3: 3 of 74, 4.1%, *p* < 0.001) and vault collaterals (IV–VI: 17 of 17, 100% versus 0 of 17, 0%, *p* < 0.001) were found more often in higher Suzuki stages compared to lower stages.

**Fig. 5:**
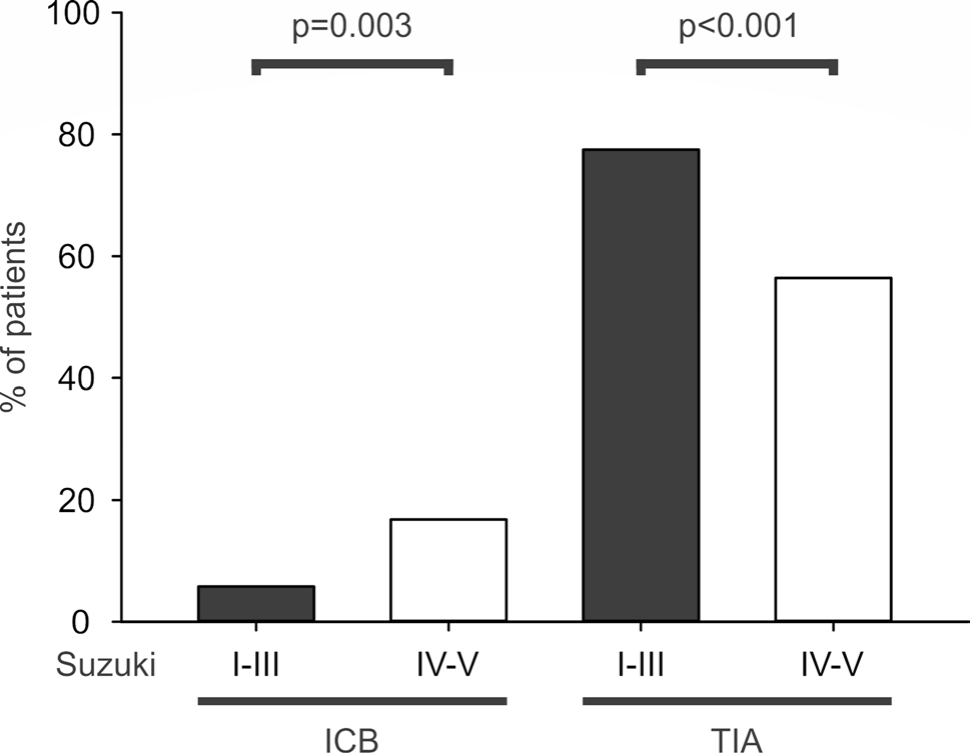
Statistical comparison of frequency of intracranial bleeding and transient ischemic attacks regarding Suzuki stages I–III versus III–IV

With regard to different Suzuki stages, we found statistical relationships in the comparisons of the frequency of cerebral hemorrhages in Pearson Chi Square tests (*p* = 0.008). More specifically, there were more intracranial hemorrhages in higher Suzuki stages, e.g. IV–VI (17 of 101, 16.8%) than in hemispheres with lower Suzuki stages, e.g. stage I–III (10 of 173, 5.8%, *p* = 0.003). In contrast to this, patients with the lower Suzuki stages I–III experienced more transient ischemic attacks (134 of 173, 77.5%) compared with the higher Suzuki stages IV–VI (57 of 101, 56.4%, *p* < 0.001) (Fig. 5). The same comparison of stroke frequency in lower versus higher Suzuki stages failed statistical significance (I–III: 147 of 173, 85.0% and IV–VI: 90 of 101, 89.1%, *p* = 0.334). Moreover, frequencies of headaches were not statistically different in patients with lower versus higher Suzuki stages (I–III: 91 of 173, 52.6% versus IV–VI: 50 of 101, 49.5%, *p* = 0.621).

Modified Suzuki stage: Out of 279 analyzable hemispheres, 55 (19.7%) were categorized as stage I, 61 (21.9%) as stage II, 145 (52%) as stage III, and 18 (6.4%) as stage IV.

Collaterals: In 32 of 280 analyzed hemispheres (11.4%), no typical collaterals were found. In 53 of 280 hemispheres (18.9%), isolated basal collaterals (pathway I) were detected. In 104 hemispheres (37.1%), choroidal and pericallosal collaterals (including basal collaterals, pathway II) were visualized. Ethmoidal collaterals (pathway III) were discovered in 74 hemispheres (26.4%). In 17 hemispheres (6.1%), vault collaterals (pathway IV) were noted. In 56 (20%) angiograms, spontaneous extra-intracranial collaterals from external carotid artery branches were present.

Pearson chi-squared tests revealed a statistical association between intracranial collaterals and the incidence of intracranial bleeding (two-sided asymptotic significance *p* = 0.003). More specifically, in patients with choroidal and pericallosal collaterals, cerebral hemorrhage occurred less frequently (3 of 104, 2.9%) compared to patients with the combined other types of intracranial collaterals (basal ethmoidal collaterals and vault collaterals) (20 of 140, 14.9%, *p* = 0.003). The patients with ethmoidal collaterals had a higher percentage of intracranial bleedings (14 of 71, 19.7%) compared with the other three types of intracranial collaterals (9 of 173, 5.2%, *p* < 0.001).

For the relationship between different types of collaterals and ischemic stroke, statistical significance was also demonstrated: patients with isolated basal collaterals had a lower frequency of stroke (41 of 52, 78.8%) compared to patients with the other types of collaterals (175 of 192, 91.1%, *p* = 0.014).

Statistical analysis found no association between different types of collaterals and occurrence of headaches (*p* = 0.090, data not shown).

Spontaneous extra-intracranial anastomoses were found more frequently in higher Suzuki (4–6: 75 of 104, 72.1%) stages compared to lower Suzuki stages (34 of 174, 19.5%, *p* < 0.001). Moreover, in patients with vault collaterals spontaneous extra-intracranial collaterals were found more often (16 of 17, 94.1%) compared to patients without vault collaterals (91 of 231, 39.4%, *p* < 0.001).

Aneurysms: In 10 of 155 patients (6.5%), the angiogram revealed a cerebral aneurysm.

Statistical analysis found no relationship between frequency of aneurysms and different localizations of collaterals (basal, choroidal and pericallosal, ethmoidal and vault) (1.9%, 6.7%, 12.2%, 5.9%, respectively, *p* = 0.171).

Posterior artery involvement: In 13 patients (8.4%), a stenotic P1 segment of the posterior cerebral artery was found.

## Discussion

Despite being a rare vasculopathy, MMA is well known among physicians in Japan and Korea and treatment regimens have been established [8]. By contrast, disease presentation and management is a matter of controversy outside Asia.

Despite the availability of excellent MRI techniques, conventional cerebral angiography is still considered as the gold standard in MMA [11]. The great advantage of conventional angiography is its exact depiction of the cerebral vasculature. Angiograms allow visual diagnosis of MMA with typical collaterals and its discrimination from other condition in the differential diagnosis [5]. These include intracranial thrombus or intracranial stenosis in varicella zoster virus-associated vasculitis or after dissections, although, in most cases, additional examinations, such as cerebrospinal fluid studies, are necessary [5]. Particularly in patients with unilateral forms, careful differential diagnostic work-up is necessary to avoid incorrect treatment.

The Suzuki grading system is well accepted [1, 12]. Whereas stage I is not specific for MMA, subsequent stages demonstrate characteristic Moyamoya collaterals and ensure the correct diagnosis. The Suzuki grading system indicates an intrinsic compensatory reorganization process from isolated stenosis at the terminal internal carotid artery (I), subsequent development of Moyamoya vessels (stages II–III), to compensatory development of trans-dural/trans-cranial anastomosis from the external carotid artery (ECA) system, and finally to disappearance of Moyamoya vessels at the late stage (stages IV–VI) and disappearance of terminal ICA [13]. Therefore, this physiological reorganization “*internal carotid (IC)-external carotid (EC) conversion*” process represents the *ideal* natural course of the disease [13]. Our analysis showed the expected highly significant distribution of collaterals according to Suzuki stages (basal, choroidal as well pericallosal collaterals in stages I-III, and ethmoidal and vault collaterals in stages IV–VI).

It can be hypothesized that, based on the Suzuki stage, a rough estimate of the duration of the disease can be made. However, the understanding of the duration, chronology and factors contributing to this process is incomplete.

In almost all angiograms in the present study, the time at which the angiograms were acquired represented the time point of diagnosis. Our study showed that most patients in this German cohort were diagnosed at Suzuki stages III and IV. It is interesting to compare these results with those from elsewhere, for example, with Eastern India where patients underwent angiography at a later stage of the disease (Germany: Suzuki stage I–III 62.7%, and stage IV–VI in 37.3% versus India: stage I–III 31.6%, and stage IV–VI 68.4%)[14]. In India, a positive correlation was demonstrated between the latency period from first symptoms to correct diagnosis and Suzuki stages (Spearman’s coefficient – 0.252, *p* = 0.028) [14]. However, even in Germany, which has a highly efficient health system, a recent study reported a high number of initial misdiagnoses (62%) and a long latency period of 5.2 years from first symptom to correct diagnosis [5]. Similar distributions of Suzuki stages to those seen in our study were found in another smaller study of 74 hemispheres from French-speaking Canada, Switzerland and France, with 58% at Suzuki stages I–III and 42% at stages IV–VI [15]. In the US, a study about children found 54% with Suzuki stages I–III and 46% with Suzuki stages IV–VI [16]. In contrast to these data, in Korea the disease seems to be diagnosed in earlier Suzuki stages, as in Korea 72% were diagnosed in stages I–III and 28% in stages IV–VI [17].

Traditionally, in Japan and Korea, it was assumed that children are at risk for TIA and ischemic strokes, whereas adults are at higher risk for intracranial bleeding [6]. This was explained with longer duration of the disease and longer presence of collaterals [6]. By contrast, this age-related symptom distribution was not confirmed in Caucasians [6]. It has been discussed whether there is a distinct western phenotype with different clinical presentation [4, 18]. However, our data demonstrated that in higher Suzuki stages (IV–VI), intracranial hemorrhages are significant more frequent than in lower stages (I–III). This is in line with recent data from the Japan Moyamoya trial, which found Suzuki staging to be significantly higher in hemorrhagic- versus ischemic-onset cases [19]. Similar results were found in patients from Korea with a strong tendency for advanced Suzuki stages in the hemorrhagic compared to the ischemic subgroup (*p* = 0.061) [17]. Moreover, our study found that TIA is significantly more common in lower Suzuki stages (I–III) versus higher Suzuki stages (IV–VI). This corresponds indirectly with Asian age-related disease presentation and indicates that missing age-related symptom distribution in studies outside Asia does not represent a different disease phenotype but might be biased by lower studied numbers [3, 5].

Despite promising new approaches using MRI to describe collaterals [20], the accuracy in conventional angiograms is still unsurpassed. Several studies have described intriguing collateral networks based on conventional angiograms [12, 21]. Such high-resolution cannot be achieved in routine 3-Tesla-MR angiography. However, in 7-Tesla MRI, which is not feasible for use in clinical routine, neither today nor in the future, very detailed microstructures could be depicted [22]. Our study described extensive collaterals and these collateral networks are known to correlate with disease severity [12]. Strother et al. demonstrated that the presence of leptomeningeal collaterals (*p* = 0.008) and dilation of the anterior choroidal artery (*p* = 0.01) was significantly correlated with disease severity [23]. Fragile vessel wall and microaneurysms of collaterals are the pathophysiological factors causing cerebral bleeding [3], which can be triggered, for example, by an increase in blood pressure. Despite the ongoing debate about differences in MMA between Western and Asian countries [4, 18, 24], an autopsy showed identical vessel wall structure as well as collateral microaneurysm in a deceased German patient [3]. Moreover, exhaustion of the collaterals is associated with a significant risk of hemodynamic stroke [7]. Vasoactive drugs or causative factors, such as hyperventilation during sexual intercourse, singing, eating spicy food, or sudden blood pressure reduction, can trigger collapse of these compensatory networks [25].

Our study demonstrated that choroidal and pericallosal collaterals are associated with significantly lower rates of intracranial hemorrhages than those found for other types of collaterals. In patients with ethmoidal collaterals, significantly more hemorrhages were observed compared to other collateral types. The Japan Moyamoya Trial found choroidal anastomoses as a factor associated with posterior hemorrhage [26]. This finding is not in conflict with our results as our ethmoidal collateral pathway III included choroidal collaterals. Moreover, the Japanese trial used a much more imprecise graduation of collaterals, only differentiating between lenticulostriate, thalamic and choroidal anastomoses [26, 27], compared to the detailed classification according to the different pathways used in our study according to Suzuki and Kodama [12]. Furthermore, a study from Korea with 175 patients found that ethmoidal collaterals were more common in hemorrhagic than in ischemic hemispheres which is in line with our results [17].

Another statistically significant relationship was found in our study for isolated basal collaterals which were associated with a lower frequency of stroke compared to those patients with the other types of collaterals.

The high incidence of spontaneous anastomoses in our study (20%) is an important finding. Spontaneous extra-intracranial anastomoses are evident in higher Suzuki (IV–VI) stages and rare in lower stages (I–III). This is congruent with the high-number study from the angiographic study from Boston Children's Hospital and Harvard Medical School [28]. This demonstrates that a full six-vessel angiogram, including visualization of the ECA, is necessary to fully understand compensating networks and to plan surgery, as damage to spontaneous anastomoses should be avoided during bypass surgery. Stimulation of nociceptors by meningeal collaterals has been proposed as the etiological factor causing headaches in patients with MMA [29]. Headaches are common (up to 67.3% of patients) [30] and partial improvement is reported after bypass surgery [30]. This could be due to regression of collaterals, which is also the reason for the decreased bleeding risk. Even though visualization of collaterals is the main reason why angiography is still considered the diagnostic gold standard, angiography is not required in the follow-up of patients after surgery, as regression of most collaterals can be demonstrated in MRI and bypass patency can easily be confirmed in Duplex ultrasound studies.

Our study found that a high proportion (77.4%) of patients had the CBN sign, which is defined as a reduction in the diameter of the extracranial internal carotid artery of 50% or more compared with the common carotid artery. The CBN sign is a correlate of a collapsed vessel due to downstream intracranial internal carotid stenosis or occlusion. To the best of our knowledge, our study is the largest study analyzing CBN systematically [31, 32]. A study in Asia found a high prevalence of CBN sign in patients with ipsilateral hemorrhage (63.7%) [32]. Awareness of this sign is important to prevent patients with MMA being misdiagnosed as having extracranial arteriosclerosis, dissection or vasculitis and receiving the wrong treatment. Differentiation of non-inflammatory CBN sign from inflammatory Takayasu arteritis is extremely important, as immunosuppressants, including corticosteroids, can have thrombotic and other serious side effects. Despite the association of MMA with thyroid autoimmune disorders, occasional contrast-enhancing vessel wall findings in black blood MRI [33], associated skin signs, such as livedo reticularis, and pathophysiologically involved autoimmune factors [34], MMA is typically not a vasculitis. Therefore, extensive differential diagnostic evaluation, including CSF studies, is advisable in cases of uncertainty [5].

Another important result of this study was the detection of posterior artery involvement and a significant proportion of associated aneurysms in this Caucasian cohort, which is in line with the existing, but limited literature. The rate of 6.5% of patents with aneurysms found in our study is comparable with Korean data (5% of patients with aneurysms) [17]. Another non-Asia study from the Stanfort School of Medicine, USA, found 3.7% of patients suffering from aneurysms [35].

Posterior stenosis was found in a high percentage (37%) of pediatric MMA patients in Japan [36]. A study from Korea detected a significantly higher percentage of posterior artery involvement in 50% of patients with RNF 213 mutations compared to 0% in patients without this mutation [37].

We are aware of some limitations of our study which make it difficult to draw generalized conclusions. One limitation is the heterogeneity of grading systems, making comparisons difficult, for example, for collaterals in addition to the traditional pathways utilized here [12, 38], as numerous other classification systems exist [23, 26, 39–41]. Another limitation is that this study is a single-center study with predominantly German patients, which also hinders generalization.

However, as data outside from Asia are rare, this study is a useful addition to the evidence base created to better characterize non-Asian MMA.

To the best of our knowledge, this study with 155 Caucasian patients and 280 angiographic hemispheres analyzed, is the largest study outside from Asia. Other studies from the USA also examined large numbers of angiograms, but in ethically heterogeneous cohorts [28] and without separate analysis by ethnicity. Therefore, the design of our study allowed us to systematically shed light on the angiographic disease presentation in non-Asian patients. In synopsis with clinical data [18] and single autopsy cases [3, 42] from non-Asian patients, our study argues for similarity of Western MMA with Asian disease presentation [4] despite other genetic backgrounds [2, 43, 44]. High estimated number of unreported cases as well later diagnosis seem to bias disease perception in Western neurologists [5]. Whether the onset of vasculopathy is really later in non-Asian patients [6, 45], and whether symptoms of MMA in childhood, such as cognitive problems and poor performance at school, are overlooked, has to be clarified. Characteristic angiographic features described here contradict the hypothesis of a more benign Western phenotype [4, 46] and may argue for similar treatment regimens as used in Asians [47].

## Data Availability

Due to protection of data privacy, angiographic data cannot be shared.
